# Parasitological, Hematological, and Immunological Response of Experimentally Infected Sheep with Venezuelan Isolates of* Trypanosoma evansi*,* Trypanosoma equiperdum*, and* Trypanosoma vivax*

**DOI:** 10.1155/2019/8528430

**Published:** 2019-02-06

**Authors:** Nereida Parra-Gimenez, Armando Reyna-Bello

**Affiliations:** ^1^Laboratorio de Fisiología de Parásitos. Centro de Biofísica y Bioquímica, Instituto Venezolano de Investigaciones Científicas, Caracas, Venezuela; ^2^Grupo de Inmunobiología, Centro de Estudios Biomédicos y Veterinarios, Universidad Nacional Experimental Simón Rodríguez-IDECYT, Caracas, Venezuela; ^3^Grupo de Investigación en Sanidad Animal y Humana (GISAH), Departamento de Ciencias de la Vida, Carrera Ingeniería en Biotecnología, Universidad de las Fuerzas Armadas ESPE, Ecuador

## Abstract

There are three trypanosoma species of veterinary importance in South America: (1)* Trypanosoma evansi,* the causative agent of derrengadera mechanically transmitted by bloodsucking insects such as tabanids, (2)* Trypanosoma vivax*, also mechanically transmitted by some dipteras hematophages as tabanids and/or* Stomoxys, *and (3)* T. equiperdum,* a tissue parasite adapted to sexual transmission and the causative agent of dourine, a distinctive disease that affects only Equidae. In order to evaluate the parasitological, hematological, and serological response of sheep infected with* T. vivax*,* T. evansi*, and* T. equiperdum*, four female sheep were experimentally infected with Venezuelan trypanosome field isolates: two* T. evansi *of differing virulences, one* T. equiperdum*; one* T. vivax*. Parasitemia and clinical parameters such as hematocrit, red blood cell count, hemoglobin, and body temperature were measured.* T. evansi* caused a chronic disease with undulant parasitemia alternating with some cryptic periods of at least 54 days, with no clinical signs.* T. equiperdum*, never described as infectious to ruminants, also caused a chronic disease with low undulant parasitemia.* T. vivax* caused an acute infection with severe anemia showing a drop of more than 70% of the hematocrit value, high fever, and rapid deterioration of physical condition, for 36 days of infection. Indirect ELISAs using crude extracts of the three species of trypanosomes as antigens were performed for detection of anti-trypanosome antibodies in sheep sera. Cross-reaction was observed between the three parasite species. These results show that sheep are susceptible to the three-trypanosome species and suggest they can act as a reservoir when sheep are raised and managed with other important livestock such as cattle, horses, buffalos, or goats. These findings are especially interesting for* T. equiperdum,* a species that has not been reported as infective to sheep.

## 1. Introduction

Haemoparasitoses represent one of the main limitations for the development of the cattle industry in Africa, Asia, and Latin America. The economic losses caused by parasitic diseases are attributed mainly to reduced weight gain, lower milk production, high mortality, and high costs of veterinary care [1, 2]. Only two species of trypanosomes with veterinary implications in South America have been identified due to their pathogenicity in livestock:* Trypanosoma vivax *and* T. evansi* [1]. However, Sanchez et al. [3] recently confirmed the first molecular report of* T. equiperdum* in Latin America.

*Trypanosoma evansi *is a mechanically transmitted extracellular blood parasite that causes equine trypanosomosis or surra, also known as Derrengadera in South America [4, 5]. Although it most notably affects horses and camels, it can also affect donkeys, dogs, cats, cattle, and buffalo [6]. Other wild mammals, such as capybaras* (Hydrochoerus hydrochaeris)*, act as reservoirs [7]. Experimental* T. evansi* infections in sheep have shown a chronic and often cryptic disease, with low parasitemias and some cases of self-cure [8–11]. Desquesnes et al. [5] review a naturally infected sheep during outbreak in France due to* T. evansi*. In South America, specifically in Brazil, there are reports of vertical transmission using experimental infection in pregnant sheep [12]. The clinical signs of* T. evansi* infection include anemia, recurrent fever, weight loss, emaciation, swelling of the hind limbs, and hemostatic abnormalities [6, 13].

*Trypanosoma vivax* is one of the most important pathogenic species of trypanosoma and is cyclically transmitted to domestic livestock by tsetse flies (*Glossina* spp,) as well as by mechanical means though other bloodsucking insects [1, 4, 14].* T. vivax* cause a critical and often fatal disease in ruminants such as cattle, buffalos, sheep and goats, due to the high fever and induced anemia [1, 15]. In Brazil, the infection by* T. vivax* has also been described in horses [16]. Clinical signs include fever, emaciation, anorexia, and immunosuppression. Thrombocytopenia, microthrombus formation, and hemorrhage suggestive of disseminated intravascular coagulation have also been demonstrated [14, 17, 18]. This haemoparasite was introduced to South America by cattle imported from Africa, as it can be transmitted by tabanids and stomoxes. Once this haemoflagelar arrived in Latin America, with the absence of Tsetse fly,* T. vivax,* it initiated an evolutionary differentiation by losing genes from its maxicircle [19]. The parasite has spread in the continent where it is now endemic from Brazil to Costa Rica, including Colombia, Venezuela, Peru, Bolivia, Panama, Paraguay, and Ecuador [1, 14, 17, 20, 21].

*T. equiperdum* is the causative agent of dourine in horses and donkeys. It differs from other mammalian trypanosomes since it is transmitted sexually and is primarily a tissue parasite, very difficult to find in blood [22–25]. Dourine has a worldwide distribution; it has been reported in Africa, Asia, Southern and Eastern Europe, Mexico, and Venezuela [3, 25].

The objective of this preliminary study was to evaluate the effect of experimental infections in sheep with three trypanosomes of veterinary interest in the region:* T. evansi*,* T. vivax*, and* T. equiperdum*.

## 2. Materials and Methods

### 2.1. Trypanosomes

One* T. equiperdum *isolate (TeAp-N/D1) and two* T. evansi *isolates (R38 and TeApMantecal01) from horse (*Equus caballus*) blood [7] and one* T. vivax* isolate (Tucacas) from cattle* (Bos taurus*) blood (kindly donated by Dr. Garcia) (Table 1) were used in this study.

### 2.2. Isolation of Trypanosomes

For* T. evansi* and* T. equiperdum* infections, the cryopreserved isolate were inoculated into the peritoneum of Sprague Dawley rats to obtain sufficient parasites for experimental sheep infections. When parasitemia reached 10^8^ trypanosomes/ml, blood was obtained by cardiac puncture in the presence of heparin as anticoagulant under anesthesia with ether. Parasites were counted using a Neubauer chamber.

### 2.3. Experimental Animals

Four healthy, helminth-free crossbreed female sheep, between 3 and 6 months of age, were obtained locally and maintained for a month prior to experimental infection. For* T. evansi* and* T. equiperdum, *fresh 1 x10^6^ parasites coming directly from infected rats were inoculated, while, for* T. vivax*, 1 x 10^6^ cryopreserved ones were injected. After inoculation, the course of the infection was monitored for 65 days for* T. evansi *and* T. equiperdum *infections, but the* T. viv*ax infected sheep was treated with 0.5 mg/kg isometamidium chloride (Hemoveex ®, Reveex, Venezuela) at 36 days postinfection to prevent animal death. The presence of parasites in the blood, rectal temperature, hematocrit, erythrocyte count, and hemoglobin concentration was monitored every other day during the experimental infection. A noninfected sheep was used as a control and the same clinical parameters were measured.

### 2.4. Detection of Trypanosomes in the Blood

For detection of parasitemia, 100 microscope fields of wet films were examined using a light microscope at 40 X magnification, as described by Brenner [26]. The parasitemia was expressed as parasites/ml and the hematocrit centrifugation technique (HCT) [27] was used to observe parasites in case of very low levels of parasitaemia.

### 2.5. Body Temperature

Every other day at the same hour and using a rectal thermometer, temperature was measured and expressed in °C.

### 2.6. Hematocrit

For determination of packed cell volume (PCV), blood (60 *μ*l) was drawn into heparinized micro capillary tubes, which were centrifuged at 10.000 g per 5 min and the PCV was measured using a microhematocrit scale.

### 2.7. Red Blood Cell Count

To determine hematological data, a sample of sheep blood was drawn, using vacuum tubes containing EDTA as anticoagulant. The blood was diluted 1: 1000 in 3% trisodic Citrate and 1% formol. Red blood cells (RBC) were counted with an optical microscope using a Neubauer chamber.

### 2.8. Hemoglobin

The cyanmethemoglobin method was used to determine hemoglobin concentration. Blood was diluted in Drabking solution and the absorbance was assessed photometrically at 540 nm on a Milton Roy Spectronic1201.

### 2.9. Serum Sample

All blood samples were collected from the jugular vein in vacuum tubes without anticoagulant. The tubes were labeled and the blood was allowed to clot overnight at room temperature and the serum was separated by centrifugation. Samples were stored at -20°C until testing.

### 2.10. Preparation of Antigen for ELISA

Parasites for* T. evansi* and* T. equiperdum* antigen preparation were obtained from experimentally infected rats as describes previously. Parasites were purified by anion exchange chromatography using DEAE-Cellulose by described Lanham and Godfrey [28]. The resultant parasites pellet was resuspended in 2 ml of PBS 20 mM, pH 7.2, and disrupted by thermal shock. The resulting homogenate was stored at -20°C with proteases inhibitors until used.

For* T. vivax *parasites purification, the protocol described by Gonzalez et al. [29] was used.

### 2.11. ELISA Procedure

For each trypanosome isolate, an ELISA plate was used, all under the same conditions, with each sample in duplicate. Negative serum samples were obtained from each sheep two weeks prior to infection and used as negative controls. During experimental infection, samples were taken at days -1, 2, 10, 18, 24, 30, 39, 47, and 61. Sensitized plates with 20 *μ*g/well of antigen were washed five times with washing buffer (WB) (PBS-Tween 0.05 %, pH 7.2) and blocked with 200 *μ*l/well of 5% skim milk in PBS for an hour at 37°C. Plates were washed five times with WB, and 100 *μ*l/well of each sera diluted 1:200 in WB was added. Positive and negative reference sera were included in each ELISA plate. After incubation for an hour at 37°C, plates were washed five times with WB, and 100 *μ*l of conjugate (Rabbit peroxidase conjugated anti-sheep IgG Pierce. Immunopure ® Antibody) diluted 1:5000 with WB was added and plates were incubated for 60 minutes at 37°C. After incubation, the plates were washed three times (PBS-Tween 0.05 %, pH 7.2) and 100 *μ*l of substrate solution, ABTS 2% H_2_O_2_ was added and incubated at 37°C for 45 min. Absorbance was measured photometrically at 405 nm on an ELISA reader (BioRad 3550).

## 3. Results

### 3.1. Disease Course

#### 3.1.1. Parasitemia

**Figure 1:**
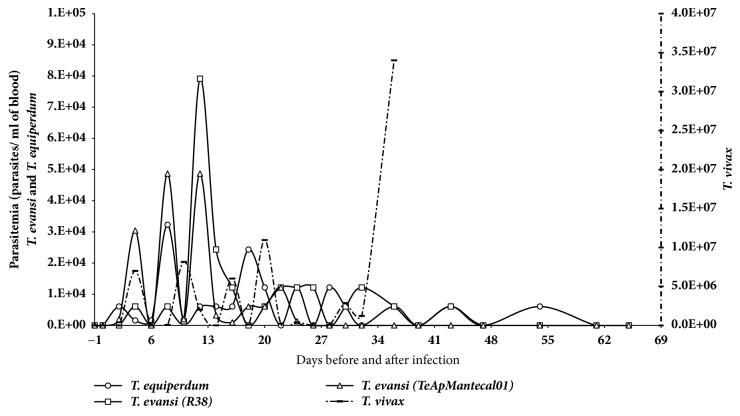
*Parasitemia score in experimental ovine infection by Trypanosoma evansi (TeApMantecal01 and R38), Trypanosoma equiperdum (TeAp-N/D1), or Trypanosoma vivax (Tucacas) isolates.* Sheep were inoculated intravenously via jugular vein with 1 x 10^6^ parasites obtained from infected rats. Parasitemia was determined by direct counting from blood using a Neubauer chamber.

Infection profiles of the* T. evansi, T. equiperdum,* and* T. vivax* isolates are shown in Table 2. Animals infected with* T. evansi* and* T. equiperdum* were monitored for 65 days, while the sheep infected with* T. vivax* was monitored until day 36 when it was treated with a trypanocide drug. Animals infected with* T. evansi *and* T. equiperdum* developed chronic trypanosomosis with fluctuating parasitemias.* T. evansi* parasitemia reached a maximum value of about 7.9 x 10^4^ tryp/ml blood (Figure 1). Parasites were detected by the Woo method [27] at 24, 43, and 54 days postinfection. In the* T. vivax* infection, parasitemia reached 3.4 x 10^6^ tryp/ml blood at day 36. During infection, it showed an undulating parasitemia ranking between 1 x 10^2^ and 5 x 10^4^. The prepatent period for all infections was 2 days (Table 2)

#### 3.1.2. Clinical Changes

**Figure 2:**
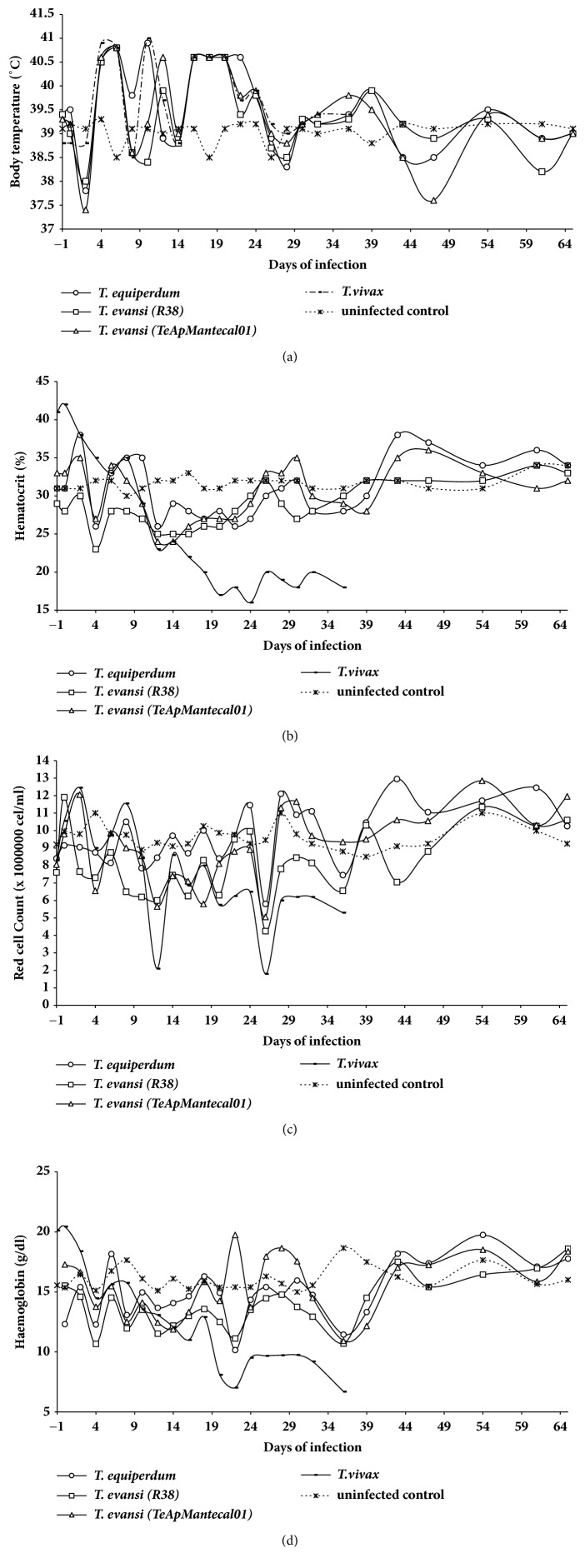
*Clinical changes in experimental sheep infection by Trypanosoma evansi (TeApMantecal01 and R38), Trypanosoma equiperdum (TeAp-N/D1), and Trypanosoma vivax (Tucacas) isolates.* Sheep were inoculated intravenously via jugular vein with 1 x 10^6^ parasites obtained from infected rats. The course of the infection was monitored for 65 days without treatment for* T. evansi *and* T. equiperdum *infections. The* T. viv*ax inoculated sheep were treated (Trypamidium ®) at 36 days postinfection to prevent animal death and safeguard its health. The clinical parameters monitored interdaily were as follows: (a) rectal temperature was determined using a column thermometer and expressed in °C. (b) Changes in hematocrit values: PCV was estimated using microhematocrit tubes every 2 days and expressed as the average percent. (c) Red cell count was determined by direct counting from blood using a Neubauer chamber and expressed in cell/ml of blood. (d) Haemoglobin concentration was determined by cyanmethemoglobin method and expressed in g/dl.

The clinical changes were evaluated during* 6*4 day*s *postinfection. There were varying increases in the body temperature that coincide with parasitemia peaks in the* T. evansi*,* T. vivax*, and* T. equiperdum *infections (Figure 2(a)). Decreases in the hematocrit values in red cell count and hemoglobin were observed from the beginning of the infection until day 22 postinfection for the* T. evansi *and* T. equiperdum *infections, afterwards values returned to the normal range (Figures 2(b), 2(c), and 2(d)). In the* T. vivax* infection, significant decreases in these parameters were observed, as compared to the noninfected or infected with the other trypanosomes.

### 3.2. Parasite-Specific Antibody Responses* (Antibody-ELISA)*

**Figure 3:**
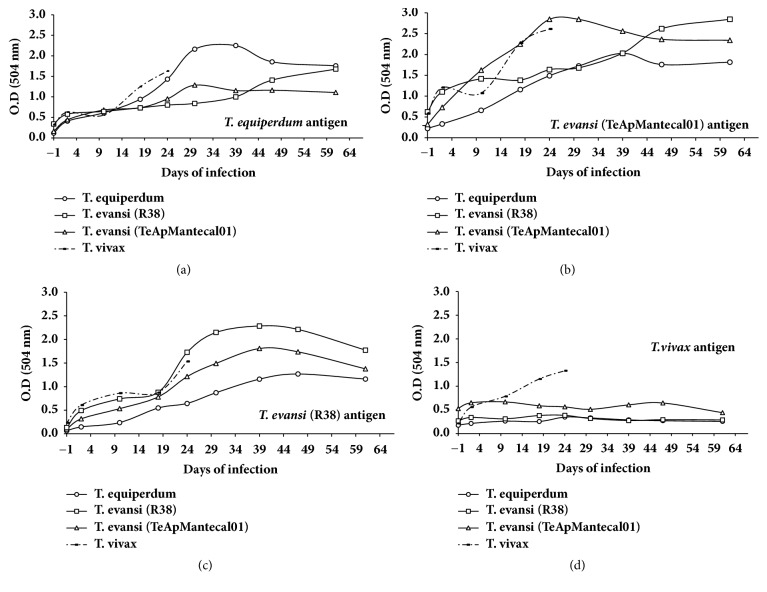
*Antibody response in serum in experimental sheep infection by Trypanosoma evansi (TeApMantecal01 and R38), Trypanosoma equiperdum (TeAp-N/D1), and Trypanosoma vivax (Tucacas) isolates.* (a) Sera from all sheep against* T. equiperdum *(TeAp-N/D1) antigen. (b) Sera from all sheep against* T. evansi *(TeApMantecal01) antigen. (c) Sera from all sheep against* T. evansi *(R38) antigen. (d) Sera from all sheep against* T. vivax *(Tucacas) antigen.

The kinetics of anti-*T. evansi, *anti-*T. vivax*, and anti-*T. equiperdum* antibodies in the experimentally infected sheep are shown in Figure 3. The ELISA tests were either homologous, in which the sera reacted to antigens from the same isolate, or heterologous, whereby sera bound antigens from a different isolate. When the sera were examined against homologous and heterologous antigens of* T. evansi, T. vivax*, or* T. equiperdum, *homologues antigens showed a greater recognition. However, for the* T. vivax *antigen, recognition of the homologous sera is higher than the heterologous system. Note that the increase in the serologic response is continuous throughout the period of the infection when heterologous system is used; however, in homologous systems there is an increase in all antibody kinetics between days 24 and 39.

## 4. Discussion

*Experimental Infection with T. evansi, T. equiperdum, and T. vivax.* In the present paper, experimental infection of sheep with* T. evansi*,* T. equiperdum*, and* T. vivax* was achieved. Data show a* T. vivax* acute infection with dramatic decrease of hematocrit and red blood cells counts. In contrast,* T. evansi* and* T. equiperdum* isolates caused a chronic disease with slight clinical manifestations.

Interestingly,* T. equiperdum* is described as the only sexually transmitted trypanosome and not as a vector borne disease. Its host spectrum is very narrow, infecting almost exclusively equine and rabbits after experimental intratesticular inoculation [25, 30]. Once these strains have been adapted to the rabbit, other rodents such as rats and mice can be infected (OIE, 2016). A reference author on trypanosomiasis [30] also points out that* T. equiperdum*, a tissue parasite, rarely invades the blood. In ruminants, experimental infections could produce weak manifestations of Dourine and cryptic or low parasitemias [8, 11, 12]. In this experimental infection, we observed that* T. equiperdum* isolate was able to infect sheep and remained by 54 days of the experimental infection. Throughout the infection, the trypanosomes were observed in blood in undulating parasitaemia and the cutaneous edema characteristic of Dourine was never evident. In view of these results TeAp-N/D1 isolate obtained from a horse naturally infected in the Venezuelan savanna shows an unknown evolutionary form of* T. equiperdum* in the new world.

*Trypanosoma evansi* has a worldwide geographical distribution; Africa, Asia, and Central and South America are the main endemic areas. In natural infections of horses, various symptoms have been reported, including intermittent fever, severe anemia, limb edema, lethargy, loss of appetite, weight loss, and other symptoms [6, 31]. The virulence of a parasite depends on several factors: (a) susceptibility of the host, (b) host ability to control the parasitemia, and (c) intrinsic parasite factors, which could lead to a better adaptation to the host. Perrone et al., 2003, reported nine Venezuelan isolates of* Trypanosoma *spp. with marked differences in virulence in mice; two of them were used in the present study: TeApMantecal01 (*T. evansi*) and TeAp-N/D1 (*T. equiperdum*) [3]. Even though both isolates showed a virulent behavior in mice and rats [32, 33], the* T. equiperdum* isolate appeared extremely virulent in both experimental models, causing an acute disease. These findings agree with observations reported by Onah et al. [8] and Audu et al. [11] in experimental sheep infections with* T. evansi* in Africa, where acute and chronic phases of the disease were observed. Campigotto et al., 2015, also reported experimental infections in sheep, with mild anemia and low parasitemia. However, fluctuating parasitemias were not observed.

Our biological data suggest that sheep are susceptible to both* T. evansi* and* T. equiperdum *isolates in Venezuela. The presence of the parasites in blood leads to great economic losses and increases the odds of the parasite transmission to other hosts.

The sheep experimentally infected with* T. vivax* showed an acute disease characterized by undulating parasitemia during the first 24 days after infection, and a rapid deterioration of the physical condition. The hematocrit values and RBC count showed a progressive reduction, until it reached a value of less of 18% at the day of the trypanocidal treatment. The clinical signs confirm the high pathogenicity and virulence of this* T. vivax* isolate in this ruminant model. This agrees with findings of naturally and experimentally infected sheep and goats reported for other* T. vivax* field isolates [17, 31, 34, 35].

In Africa and America, cattle and buffalo livestock share their habitats with herds of sheep and goats. Epidemiological data of field-infected herds showed the likelihood of* T. vivax* transmission between asymptomatic carriers such as buffalo and donkeys to susceptible hosts, in a same area [34]. In Brazil, where sheep and goats are important livestock in the semiarid region, those animals may be severely affected by* T. vivax *infection, showing anemia, hyperthermia, enlarged lymph nodes, and progressive weight loss [31].

Even though we used a reduced number of experimental animals, the sheep susceptibility to* T. evansi, T. equiperdum*, and* T. vivax *was demonstrated and could open the possibilities of mixed infection in natural hosts of endemic areas due to the presence of the vector and the proximity of grazing areas.

*Antigenic Cross-Reactivity by ELISA.* The cross-reactivity between* T. evansi* and* T. vivax* is well documented (Reyna-Bello et al. [36], Desquesnes et al. [37], Uzcanga et al. [38], and Camargo et al. [39, 40]); our results show the same behavior with* T. equiperdum*. However, a reciprocal cross-reactivity does not occur when* T. vivax *is used as the antigen to detect antibodies against* T. evansi or T. equiperdum*. As this is a preliminary account of the infection of* T. equiperdum* in an ovine model, we can only speculate about the ability of the parasite to stimulate the immune response and more studies are needed to evaluate this phenomenon.

## 5. Conclusion

The experimental results of this study suggest the possibility of widespread infection by* T. evansi, T. equiperdum*, or* T. vivax* in Venezuela herds sheep. The existence of the disease should be suspected when clinical signs common to any of the three trypanosomes species appear. We state the possibility that sheep act as a reservoir for these three trypanosomes in areas where they live close to equines and cattle. In addition, it was observed in indirect ELISA that cross-reactivity between* T. evansi *and* T. equiperdum* versus* T. vivax* is curiously unidirectional.

Finally, we wish to point out that a sheep infected with a strain of* T. equiperdum,* described in previous studies, remained infected at least for 54 days, which represents an unusual finding.

## Figures and Tables

**Table 1:** Isolates of *T. evansi, T. equiperdum*, and *T. vivax* used in this study. Natural host and geographic localities. N/D: not determined.

Specie	Isolate	Natural host	Locality
*T. evansi*	TeApMantecal01	Horse	Mantecal, Apure State

*T. evansi*	R38	Horse	N/D. Apure State

*T. vivax*	Tucacas	Cattle	Tucacas, Estado Falcon

*T. equiperdum*	TeAp-N/D1	Horse	N/D. Apure State

**Table 2:** Biological data of *T. evansi, T. equiperdum*, and *T. vivax* isolates during the experimental infection in sheep. *∗∗* trypanocidal treatment was applied to the sheep.

Specie	Isolate	Prepatent period (Days)	Average parasitemia (x 10^4^ tryps/ml blood)	Period of infection (Days)	Average period of the maximal parasitaemia (Days)
*T. evansi*	TeApMantecal01	2	4,8	43	2

*T. evansi*	R38	2	7,9	24	5

*T. vivax*	Tucacas	2	34000	36*∗∗*	36

*T. equiperdum*	TeAp-N/D1	2	3,0	54	8

## Data Availability

The data used to support the findings of this study are available from the corresponding author upon request.
